# Evaluation of a Brown Seaweed Extract from *Dictyosiphon foeniculaceus* as a Potential Therapeutic Agent for the Treatment of Glioblastoma and Uveal Melanoma

**DOI:** 10.3390/md18120625

**Published:** 2020-12-08

**Authors:** Philipp Dörschmann, Christina Schmitt, Kaya Saskia Bittkau, Sandesh Neupane, Michael Synowitz, Johann Roider, Susanne Alban, Janka Held-Feindt, Alexa Klettner

**Affiliations:** 1 Department of Ophthalmology, University Medical Center Schleswig-Holstein UKSH, Campus Kiel, D-24105 Kiel, Germany; johann.roider@uksh.de (J.R.); alexakarina.klettner@uksh.de (A.K.); 2 Institute of Anatomy, Kiel University, D-24118 Kiel, Germany; christina.schmitt.bingen@gmx.net; 3 Pharmaceutical Institute, Kiel University, D-24118 Kiel, Germany; kbittkau@pharmazie.uni-kiel.de (K.S.B.); sneupane@pharmazie.uni-kiel.de (S.N.); salban@pharmazie.uni-kiel.de (S.A.); 4 Department of Neurosurgery, University Medical Center Schleswig-Holstein UKSH, Campus Kiel, D-24105 Kiel, Germany; michael.synowitz@uksh.de (M.S.); janka.held-feindt@uksh.de (J.H.-F.)

**Keywords:** fucoidan, cancer, VEGF, gene expression, toxicity, *Dictyosiphon foeniculaceus*, retinal pigment epithelium, glioblastoma, astrocytes, uveal melanoma

## Abstract

Ingredients of brown seaweed like fucoidans are often described for their beneficial biological effects, that might be interesting for a medical application. In this study, we tested an extract from *Dictyosiphon foeniculaceus* (DF) to evaluate the effects in glioblastoma and uveal melanoma, looking for a possible anti-cancer treatment. We investigated toxicity, VEGF (vascular endothelial growth factor) secretion and gene expression of tumor and non-tumor cells. SVGA (human fetal astrocytes), the human RPE (retinal pigment epithelium) cell line ARPE-19, the tumor cell line OMM-1 (human uveal melanoma), and two different human primary glioblastoma cultures (116-14 and 118-14) were used. Tests for cell viability were conducted with MTS-Assay (3-(4,5-Dimethylthiazol-2-yl)-5-(3-carboxymethoxyphenyl)-2-(4-sulfophenyl)-2H-tetrazolium), and the proliferation rate was determined with cell counting. VEGF secretion was assessed with ELISA (enzyme-linked immunosorbent assay). The gene expression of VEGF receptor 1 (VEGFR1), VEGF receptor 2 (VEGFR2) and VEGF-A was determined with real-time qPCR (quantitative polymerase chain reaction). DF lowered the cell viability of OMM-1. Proliferation rates of ARPE-19 and OMM-1 were decreased. The VEGF secretion was inhibited in ARPE-19 and OMM-1, whereas it was increased in SVGA and 116-14. The expression of VEGFR1 was absent and not influenced in OMM-1 and ARPE-19. VEGFR2 expression was lowered in 116-14 after 24 h, whereas VEGF-A was increased in 118-14 after 72 h. The extract lowered cell viability slightly and was anti-proliferative depending on the cell type investigated. VEGF was heterogeneously affected. The results in glioblastoma were not promising, but the anti-tumor properties in OMM-1 could make them interesting for further research concerning cancer diseases in the human eye.

## 1. Introduction

Tumor diseases and malignant cancer is a huge challenge and an active research field in medical treatment developments. Cancer is one of the deadliest diseases in industrial nations and a wide variety of cancer types exist. One of these cancer types is glioblastoma multiforme (GBM), one of the most dangerous and most aggressive tumor diseases, which accounts for more than 15% of all intracranial tumors and has a median survival time of 7–15 months from the time of diagnosis [1]. The probability of developing this tumor increases with age. Symptoms of the disease are nausea, vomiting, seizures and severe headaches. The incidence rate is ranges up to 3.69 per 100,000 persons [2]. Standard treatment options include surgery, radiation, and chemotherapy with temozolomide [3]. However, there are still no curative treatment concepts available. Rapid growth and early migration of the tumor cells are responsible for its poor prognosis [4]. The main intraocular cancer type is uveal melanoma (UM) with an incidence of 5.1 per million per year [5]. It generates from melanocytes located in the uvea, most commonly from the choroid. The main problem of this cancer is the potential to metastasize and spreading into the liver which leads often to death [6]. Common harsh therapies like surgery often result in vision loss in the affected eye, while no effective treatment for metastatic tumors are available [7].

The potential of natural marine substances is widely known for the beneficial uses for human health. The regular consummation of algae has been described to be correlated with a longer and healthier life, which is also described for the Okinawa diet [8]. Several marine plants like brown algae, vertebrates like fish and invertebrates contain different agents which are beneficial for the human health [8]. Especially the potential of brown algae extracts, which contain positive ingredients like polyphenols, alginates and sulfated fucans (fucoidans) is often described in the literature [8,9,10,11]. Among these positive effects are anti-oxidative, anti-inflammatory, anti-tumor and anti-angiogenic properties [12,13,14,15], making it highly interesting for cancer and skin therapies [16,17]. Fucoidans have also been described by our group for their anti-oxidative, VEGF (vascular endothelial growth factor) lowering and binding effects [18,19,20,21]. In general they do not lower cell viability as tested in different cell types like ARPE-19 (human retinal pigment epithelium), HL-60 (acute myeloid leukemia), Raji (Burkitt lymphoma), HeLa (cervix carcinoma), A-375 (skin melanoma), HCT-116 (colon carcinoma), Hep G2 (hepatocellular carcinoma) and HaCaT (keratinocytes) after 24 h of treatment [22]. However, in the uveal melanoma cell line OMM-1, certain brown algae extracts above certain concentrations decreased the cell viability [19,22]. Such extracts may be of interest in tumor diseases, such as uveal melanoma or glioblastoma.

Among fucoidan containing extracts from six different brown algae species, that from *Dictyosiphon foeniculaceus* (DF) showed the most pronounced anti-proliferative effects in tumor cell lines OMM-1 after 24 h and in HeLa after 24 and 72 h of treatment [22]. DF, also known as tubular net weed or golden sea hair, belongs to the order of Ectocarpales and is a highly branched brown seaweed species that grows on rocks, other algae, or is free-floating in North Atlantic and Northwest Pacific [23]. Literature on fucose containing sulfated polysaccharides from DF is so far limited to the mentioned DF extract [24,25]. Chemical characterization of the DF extract revealed that the applied fucoidan extraction and purification procedure did not result in fucoidans as known for many other brown algae, but a complex mixture of fucose containing polysaccharides and proteoglycans, respectively, with a relatively low sulfate content [24]. Typical fucoidan activities including antioxidant, elastase inhibiting, anticomplement, and anticoagulant effects turned out to be weaker than those of other fucoidans [24], but contrary to other fucoidans, the DF extract reduced the cell viability of two tumor cell lines [22].

In this work we tested an extract from DF in therapeutically relevant concentration range in terms of cell viability, proliferation, regulation of VEGF secretion and gene expression of VEGF-A, FLT-1/VEGFR1 (fms related receptor tyrosine kinase 1/VEGF receptor 1) and KDR/VEGFR2 (kinase insert domain receptor/VEGF receptor 2) after short- and mid-term time period stimulation of the tumor cell line OMM-1 and the human primary glioblastoma cultures 116-14 and 118-14. As a comparison, we used the healthy cells of ARPE-19 and human astrocytes SVGA. This study is well-equipped for investigation of the currently unknown effects of DF extract on tumor relevant properties like angiogenesis, cell viability and proliferation and to make a comparison between two non-tumor and three tumor cell lines as well as between two ocular and three brain cell cultures.

## 2. Results

### 2.1. Chemical Characterization of the Extract

The extraction and purification of DF was performed by a procedure usually resulting in relatively pure fucoidans. The chemical analysis of the DF extract (Table 1) conducted by Bittkau et al., 2020 and Neupane et al., 2020 [24,25] revealed that the DF extract strongly differs from the fucoidans obtained from five other brown algae species and cannot be considered as typical fucoidan. Compared to other five extracts, its sulfate content (8.8% vs. 12.3–28.8%), which resulted in a calculated apparent degree of sulfation of only 0.1, and its fucose content (38.7% vs. 40.6–96.1%) were the lowest, whereas its protein content (24.1% vs. 1.9–15.3%) was the highest (Table 1). Also the contents of other monosaccharides were considerably higher than those of the other five fucoidans, namely 32.0% xylose, 16.2% galactose and 5.6% mannose. The average molecular weight of the DF extract was 194 kDa (vs. 188–1340 kDa) with a quite high polydispersity of 3.9, its contents of glucose (5.0%) and uronic acids amounted to 98% and its content of phenolic compounds was 2.2–4.5 times lower than that of the three *Fucus* fucoidans. According to analysis by size exclusion chromatography with multiple detection, the main fraction of the DF extract had a compact spherical conformation, whereas typical fucoidans exist as random coil with expanded structure. This conformation is in line with the high protein content and suggests polysaccharides tightly associated with protein (glycoproteins, proteoglycans) [25]. Especially due these pronounced structural differences of the DF extract, it was of interest to investigate its effects on VEGF secretion, gene expression as well as cell viability and proliferation of tumor cells.

### 2.2. Cell Viability Assay

**Figure 1:**
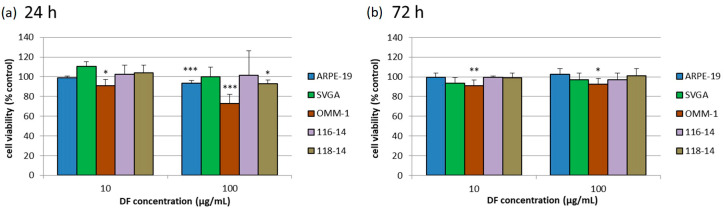
Cell viability of five different cell cultures was investigated, namely the human RPE (retinal pigment epithelium) cell line (ARPE-19), human, uveal melanoma cell line OMM-1, human fetal astrocytes SVGA and human glioblastoma cells 116-14 and 118-14. MTS-Assay (3-(4,5-Dimethylthiazol-2-yl)-5-(3-carboxymethoxyphenyl)-2-(4-sulfophenyl)-2H-tetrazolium) was performed after treatment with 10 or 100 µg/mL DF (*Dictyosiphon foeniculaceus*) extract for 24 (**a**) or 72 h (**b**). The mean values and standard deviation were calculated in relation to the untreated control (set to 100%, not shown). Significance tests were performed with One-Way ANOVA with multiple comparison test; * *p* < 0.05, ** *p* < 0.01, *** *p* < 0.001 compared to control group (*n* ≥ 4; number of independent experiments).

Cell viability of the five different cell types was assessed with the commercially available MTS (3-(4,5-Dimethylthiazol-2-yl)-5-(3-carboxymethoxyphenyl)-2-(4-sulfophenyl)-2H- tetrazolium) assay by Promega Corporation. Cells were treated for 24 and 72 h. Cell viability of SVGA, 116-14 and 118-14 was unaffected (Figure 1) except for 118-14; here 100 µg/mL DF slightly lowered the viability to 93% ± 4% after 24 h of treatment (*p* < 0.05). ARPE-19 viability was decreased after the application of 100 µg/mL of the DF extract for 24 h to 94% ± 3% (*p* < 0.001). The DF extract showed slight anti-proliferative effects in the OMM-1 cell line. In detail, 10 µg/mL DF lowered the viability of OMM-1 cells to 91% ± 6% (*p* < 0.05), and 100 µg/mL DF lowered it to 73% ± 9% (*p* < 0.001) after 24 h. Contrary, 10 µg/mL DF reduced OMM-1 cell viability to 91% ± 6% (*p* < 0.01), and 100 µg/mL DF reduced it to 93% ± 5% (*p* < 0.05) after 72 h.

### 2.3. Cell Proliferation Test

**Figure 2:**
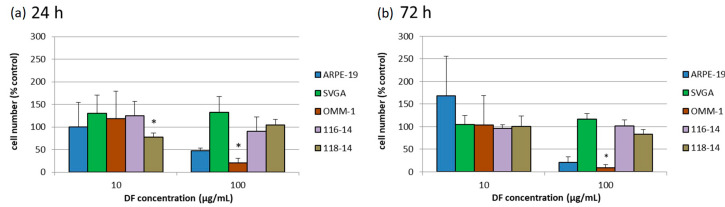
Cell proliferation of ARPE-19, SVGA, OMM-1, 116-14 and 118-14 was investigated by cell counting with trypan blue staining after treatment with 10 or 100 µg/mL DF extract for 24 (**a**) and 72 h (**b**), respectively. The mean values and standard deviation represent the mean of the cell number in relation to an untreated control in percent (set to 100%, not shown). Significance tests were performed with One-Way ANOVA with multiple comparison test; * *p* < 0.05, compared to untreated control group (*n* ≥ 4).

The influence of the DF extract on cell proliferation was determined with cell counting after trypan blue staining. The diagrams represent the relative cell number in percent to the untreated control (Figure 2). The cell numbers of ARPE-19, SVGA and 116-14 were not significantly influenced. After 24 h of treatment, the cell number of 118-14 was reduced to 77 ± 10% (10 µg/mL DF) (*p* < 0.05). An application of 100 µg/mL DF lowered the cell number of OMM-1 to 21 ± 10% (*p* < 0.05) after 24 h and to 9 ± 7% (*p* < 0.05) after 72 h of stimulation. These lowered proliferation rates of OMM-1 correspond to the lowered cell viabilities as described in Section 2.2.

### 2.4. VEGF Secretion

**Figure 3:**
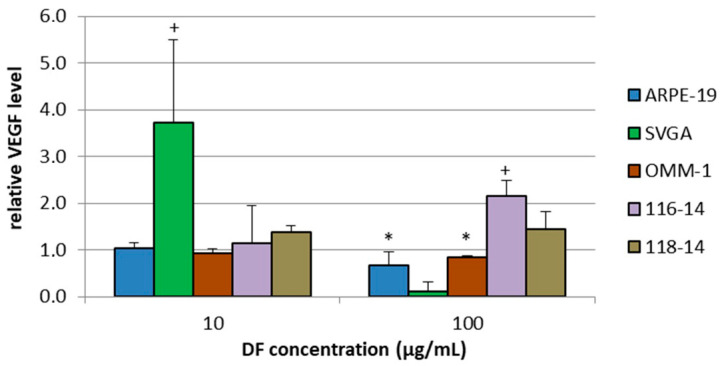
VEGF (vascular endothelial growth factor) secretion of ARPE-19, SVGA, OMM-1, 116-14 and 118-14 was assessed with ELISA (enzyme-linked immunosorbent assay) after treatment with 10 or 100 µg/mL DF extract for three days. The secreted individual VEGF amount in pg/mL was set in relation to the corresponding cell viability, which was determined in parallel. The mean values and standard deviation represent the mean of this calculated relative VEGF amount. Significance tests were performed with One-Way ANOVA with multiple comparison test; */+ *p* < 0.05, compared to untreated control group (*n* ≥ 4).

The secreted VEGF amounts of ARPE-19, SVGA, OMM-1, 116-14 and 118-14 were determined with ELISA (enzyme-linked immunosorbent assay). The individual VEGF amount in pg/mL was set in relation to the corresponding cell viability in % (cell viability assay performed, data not shown), compared to controls, to exclude the dependency of the measured VEGF secretion on the cell viability and number. The normalized, relative VEGF amounts compared to untreated controls are depicted in Figure 3. After 10 µg/mL DF application, SVGA cells responded with a high increase of VEGF secretion up to 3.7 [a.u.] ± 1.8 [a.u.] (arbitrary units) (*p* < 0.05). Additional effects could be determined after 100 µg/mL DF treatment: The secreted VEGF amount of 116-14 was increased to 2.2 [a.u.] ± 0.3 [a.u.] (*p* < 0.05), whereas VEGF of ARPE-19 and OMM-1 was decreased to 0.7 [a.u.] ± 0.3 [a.u.] (*p* < 0.05) and 0.8 [a.u.] ± 0.0 [a.u.] (*p* < 0.05), respectively. Thus, it seems that DF extracts can promote pro-angiogenic effects in brain cells and small anti-angiogenic effects in ocular cells.

### 2.5. Gene Expression

**Figure 4:**
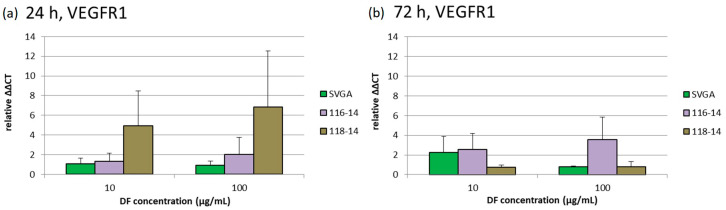
Relative gene expression of VEGFR1 (VEGF receptor 1) was determined with qPCR (quantitative polymerase chain reaction) after 24 (**a**) and 72 h (**b**) of treatment with 10 or 100 µg/mL DF extract. The potentiated ΔΔCT values are determined in relation to the corresponding untreated controls, respectively (control = 1). Significance tests were performed with One-Way ANOVA with multiple comparison test, compared to the untreated control group (*n* ≥ 4).

**Figure 5:**
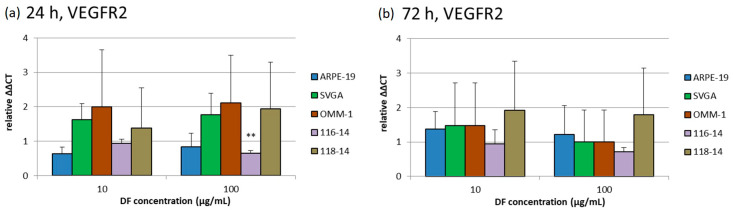
Relative gene expression of VEGFR2 (VEGF receptor 2) was determined with qPCR after 24 (**a**) and 72 h (**b**) of treatment with 10 and 100 µg/mL of the DF extract. The potentiated ΔΔCT values are determined in relation to the individual untreated controls, respectively (control = 1). Significance tests were performed with One-Way ANOVA with multiple comparison test; ** *p* < 0.01, compared to untreated control group (*n* ≥ 4).

**Figure 6:**
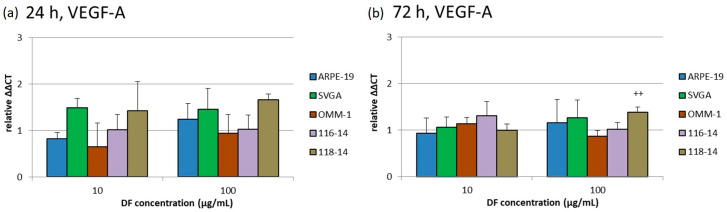
Relative gene expression of VEGF-A was determined with qPCR after 24 (**a**) and 72 h (**b**) of treatment with 10 or 100 µg/mL DF extract. The potentiated ΔΔCT values are determined in relation to the individual untreated controls, respectively (control = 1). Significance tests were performed with One-Way ANOVA with multiple comparison test; ++ *p* < 0.01, compared to untreated control group (*n* ≥ 4).

The effects of 10 and 100 µg/mL DF extract on the gene expression of VEGFR1, VEGFR2 and VEGF-A were determined with qPCR (quantitative polymerase chain reaction) after 24 and 72 h. The diagrams in Figure 4, Figure 5 and Figure 6 depict the potentiated ΔΔCT values, which are determined in relation to the corresponding untreated controls, respectively (control = 1). Expression of VEGFR1 could not be determined in ARPE-19 or OMM-1. In addition, the DF extract had no significant influence on the VEGFR1 expression of SVGA, 116-14 and 118-14.

VEGFR2 was expressed in all five cell cultures. There were no significant effects of the DF extract on the gene expression of VEGFR2 after 24 and 72 h of treatment, respectively, with one exception: the application of 100 µg/mL of the DF extract lowered the relative VEGFR2 expression in 116-14 to 0.66 [a.u.] ± 0.07 [a.u.] after 24 h. Although a statistical significance of this effect was determined, the biological relevance of this very small reduction of VEGFR2 in 116-14 must be considered critically.

VEGF-A was expressed in all five cell types. Only 100 µg/mL DF increased the relative VEGF-A expression in 118-14 to 1.66 [a.u.] ± 0.13 [a.u.]. Despite the statistical significance of this effect, the very small increase of VEGF-A expression in 118-14 has to be considered to be of little biological relevance.

## 3. Discussion

The DF extract investigated in this study strongly differs from other brown algae extracts usually consisting of fucose-rich sulfated polysaccharides. The main aim of this study was to evaluate potential anti-tumor effects of the DF extract on uveal melanoma and glioblastoma cell lines. For this purpose, we tested the effects of 10 and 100 µg/mL DF extract on the non-tumor cell lines ARPE-19 and SVGA as well as on the tumor cell line OMM-1, and the primary tumor cells 116-14 and 118-14. A first indication of anti-tumor effects of DF was described by Bittkau, Dörschmann et al., 2019. Here, 100 µg/mL DF extract reduced the cell viability of OMM-1 to nearly 75% after 24 h of treatment [22]. Futhermore, the cell viability of the cervical cancer cell line HeLa was reduced to 61–69% after 24 h, and to 70% after 72 h [22]. This corresponds to the data of this work where the OMM-1 viability was also reduced to 73% after 24 h, whereas the viability regenerated after 72 h to over 90%. Although DF lowered the viability of 118-14, this effect was quite small. The data of the viability assay also corresponds partially to the proliferation rate in case of OMM-1. In contrast to viability, ARPE-19 proliferation was not significantly inhibited and the cell number of 118-14 was reduced with a different concentration than in the cell viability assay. However, the small effects on the viability of 118-14 seem to be of little biological relevance. Regarding the effects on the gene expression of VEGF-A and its receptors, VEGFR1 and VEGFR2, only slightly increased or reduced expression levels could be detected. In detail, for VEGFR1 no significant effect was seen in the brain cells and for ARPE-19 and OMM-1 no expression of VEGFR1 was determined, which is in contrast to the literature for ARPE-19 [26] and could depend on the passage number or mutation level of these cells. An only very slight decrease of VEGFR2 expression was detected for 116-14 and a very small increase for VEGF-A was determined for 118-14. Since VEGF-A is well known to promote tumor progression [27], even a small effect is not desirable as an anticancer effect. However, an influence of fucoidans on the gene expression level is shown for commercially available fucoidan from *Undaria pinnatifida* (Marinova), which is able to change the gene expression of cancer-related and cell surface signaling-related pathways [28]. But this alga is from another brown algae order and was used in a clinical study with human blood serum, which could explain the differences. Nevertheless, the DF extract used in our study did not influence the expression of VEGF-A, VEGFR1 and VEGFR2 in our experimental setting.

So far, there are no further data or studies about cellular effects of extracts from the brown algae DF. However, a few other species of the order of Ectocarpales are described to contain fucoidans. Among these, fucoidans from *Cladosiphon okamuranus* were shown to exhibit anti-tumor effects after oral administration in a colon cancer mouse model by slowing down tumor growth and increasing the survival time depending on the molecular weight of the fucoidan [29]. This effect is suspected to correlate with an activation of the colon-associated immune cells [29].

In contrast, there are numerous reports on various anti-tumor effects of fucoidans from brown algae belonging to the orders of Laminariales and Fucales. For example, fucoidans from Fucus vesiculosus and Laminaria japonica, which are also commercially available, are often described to exhibit anti-tumor activities [30,31,32,33,34,35,36,37]. We previously found that any potential anti-tumor effects depended on the fucoidan source and the specific extract, respectively [18,19]. Middle- and low-molecular weight fucoidans from Laminaria hyperborea lowered the cell viability of OMM-1 cells [19], whereas enzymatically treated extracts from Laminaria digitata, Fucus distichus subsp. evanescens as well as various extracts from Saccharina latissima with different fucose content and degree of sulfation did not exhibit any anti-tumor effects on OMM-1 [20]. Dithmer et al., 2017 tested the effects of Fucus vesiculosus fucoidan from Sigma Aldrich on five different uveal melanoma cell types and on the one hand, this fucoidan had an anti-proliferative effect on the primary uveal melanoma cells Mel270, but not on the OMM-1 cells [38]. On the other hand, it decreased only the VEGF secretion by OMM-1 cells after three-day stimulation with the fucoidan [38]. The latter effect is consistent with the present results showing that DF extract reduced the VEGF secretion also by about 20%. However, in contrast to the Fucus vesiculosus fucoidan, the DF extract additionally had an antiproliferative effect on the OMM-1 cells. Thus, the two test compounds differed in their activities, which is probably due to their considerably different structural composition. Compared to the fucoidan, the DF extract has a more than two-fold lower sulfate and fucose content, but is characterized by 24.1% proteins [24,39]. These proteins were shown to be tightly associated with the glycans [25] as previously found for certain fractions of other fucoidans [40,41]. Since the DF extract displayed much weaker effects than other fucoidans in various activity assays (i.e., elastase inhibiting, anticomplement, and anticoagulant activities) [24], it can be assumed that its similar result in the VEGF secretion assay is mediated by molecules structurally different from those in the Fucus vesiculosus fucoidan and possibly also by a different mechanism. Regarding the antiproliferative activity of DF extract on OMM-1, it is known that the total phenolic content of fucoidans correlates with both their cell viability reducing effect and antioxidant capacity [18,39,42,43]. However, the DF extract has only a low content of phenols compared to other fucoidans [25], which is in line with its low antioxidant capacity [24]. Consequently, also its antiproliferative effect seems to be caused by other components. Comprehensive and challenging further analyses are needed to get more information on the structure of the glycan-protein-associates of the DF extract.

Regarding the effects of algae-derived substances on glioblastoma cells, knowledge is still limited. Nevertheless, studies with pheophorbide (chlorophyll breakdown product) from the red seaweed Grateloupia elliptica showed anticancer effects in U87MG cells (a human glioma cell line) by inducing G0/G1 cell cycle arrest, apoptosis and DNA degradation [44]. Further, nano-micro particles loaded with microalgae from Chlorella protothecoides and Nannochloropsis oculata had cytotoxic effects on human A-172 glioblastoma cells and HCT-116 (human colon colorectal carcinoma), whereas HUVEC (human umbilical vein endothelial cells) were not influenced [45]. It was speculated that microalgae contain anti-proliferative and apoptotic compounds and therefore, represent a source for the development of potential therapeutics [45]. Liao et al., 2019 showed that oligo-fucoidan from brown seaweed markedly suppressed the proliferation of U87MG cells and also regulated the gene expression of several differentiation markers [46]. Interestingly, Lv et al., 2012 stated that conditioned media taken from fucoidan-pretreated T98G glioblastoma cells inhibited endothelial cell tube formation leading to the assumption that at least *Fucus vesiculosus* (Sigma Aldrich) significantly inhibits angiogenesis induced by glioma cells. An up-regulation of sFlt-1 played an important role in this process [47]. In the presented study, however, relevant anti-proliferative effects on glioblastoma cells were absent, and DF extract even increased the VEGF secretion by 116-18 cells. These results might be due to the used experimental set-up, glioblastoma cell types and/or the test compound.

In general, this study again confirms the exact effects of fucoidans and brown algae extracts, respectively, on the reactions of tumor cell lines are highly dependent on the used cell line as well as on the structural composition and characteristics of the tested compound [22]. Thus, each fucoidan or brown algae extract has to be examined for each individual purpose.

Furthermore, the question of pharmaceutical applications for a possible tumor treatment is of high relevance. The bioavailability after oral administration of fucoidan is still under investigation [48,49,50,51]. However, different studies described that fucoidans can be taken up depending on the kind of application and the chemical characteristics. A *Fucus vesiculosus* fucoidan of 735 kDa in ointments can be applied topically and penetrate the skin reaching the blood plasma [50]. The same fucoidan was also detected in kidney, spleen and liver after oral administration of rats with long absorption and blood circulation times [49]. Furthermore, Japanese researches detected a high molecular weight fucoidan (3200 kDa) from *Okinawa mozuku* in urine after oral administration in human and rats [51]. Nevertheless, reaching clinical concentration in the desired tissue is a challenge and carrier/delivery systems for fucoidans would be of high interest.

In this study, we examined an extract from the brown alga DF with a composition different from that of typical fucoidans, for its potential anti-tumor effects in uveal melanoma and glioblastoma cells. However, it had no influence on glioblastoma cells, it showed some activities in the experiments with the uvea melanoma cell line OMM-1 by lowering the cell viability and exhibiting anti-proliferative and anti-angiogenic effects. The absence of negative effects on healthy cells like ARPE-19 and SVGA can be considered as beneficial, too.

Currently, there is no medicinal product approved for the treatment of uveal melanoma, the most important tumor disease of the eye, and thus a medical need for new therapies. Although the basic experiments with the DF extract look promising, this extract as well as fucoidans are associated with some obstacles regarding medical applications. This includes their complex composition and the strong requirements on the pharmaceutical quality of drug substances as well as the biopharmaceutical properties of these negatively charged macromolecules. For the treatment of uveal melanoma intravitreal application would also be of interest.

## 4. Material and Methods

### 4.1. Extraction and Chemical Characterization

The DF alga was harvested in May 2017 in the Baltic Sea (Kiel Fjord) and provided by Coastal Research and Management GmbH (Kiel, Germany). Extraction and purification was performed as described before [40]. In short: The pulverized algal material was defatted with Soxhlet extraction (99% *v/v* ethanol). The main extraction was performed with aqueous 2% CaCl_2_ at 85 °C for 2 h (reflux condition). The supernatant was evaporated and precipitated with ethanol (final concentration 60% *v*/*v*) at 4 °C. Further steps involved centrifugation, dissolving in demineralized water, dialysis and lyophilisation. The following chemical parameters of the DF extract were analyzed as previously described [24,25]: Sulfate content and degree of sulfation, weight average molecular weight, monosaccharide composition, contents of protein, uronic acid content, and phenolic compounds as well as size distribution and chain conformation.

The dried extract was solved in Ampuwa bidest. (Fresenius, Schweinfurt, Germany) to a stock concentration of 1 mg/mL. Before use in experiments, the stock solution was diluted to 100 µg/mL in appropriate medium, sterile filtered with 0.2 µm Sarstedt filters (Nümbrecht, Germany) and further diluted with medium to 10 µg/mL. Final medium concentrations were 10 and 100 µg/mL in each case. The final medium volume was 1 mL per well in 12 well plates (Section 2.3, Section 2.4 and Section 2.5 assays) and 100 µL per well in 96 well plates (Section 2.2), respectively.

### 4.2. Cell Culture and Reagents

Used cell lines were the uveal melanoma cell line OMM-1 [52], the human RPE cell line ARPE-19 [53], the human fetal astrocyte cell line SVGA and the human glioblastoma (GBM) primary cells 116-14 and 118-14. ARPE-19 was purchased from ATCC (ATCC, Manassas, VA, USA). OMM-1 was kindly provided by Dr. Sarah Coupland, University of Liverpool. The human fetal astrocyte cell line SVGA was kindly provided by the group of Christine Hanssen Rinaldo, University Hospital of North Norway [54] with the permission of W. J. Altwood [55]. RPMI 1640 (Merck, Darmstadt, Germany), which was supplemented with 10% fetal calf serum (Linaris GmbH, Wertheim-Bettingen, Germany) and 1% penicillin/streptomycin (Merck), was used for OMM-1. Cultivation medium for ARPE-19 was HyClone Dulbecco’s modified Eagle’s medium (DMEM; GE Healthcare, München, Germany), with 10% fetal calf serum, 1% penicillin/streptomycin, 2.5% HEPES (4-(2-hydroxyethyl)-1-piperazineethanesulfonic acid, Merck) and 1% non-essential amino acids (Merck). Cultured human primary GBM cells were generated by dissociation of surgically dissected tumor materials and cultured in DMEM (Life Technologies, Carlsbad, CA, USA) supplemented with 10% fetal bovine serum (FBS; Invitrogen, Carlsbad, CA, USA or PAN-Biotech GmbH, Aidenbach, Germany), 1% Penicillin–Streptomycin (10,000 U/mL; Thermo Fisher Scientific, Waltham, MA, USA), and 2 mM additional L-glutamine (Thermo Fisher Scientific). Materials were obtained in accordance with the Helsinki Declaration of 1964 and its later amendments and with approval of the ethics committee of the University of Kiel, Germany after written informed consent of donors (file references: D571/15 and D524/17). Tumors were diagnosed and classified according to WHO criteria by a pathologist. All cell lines were stored at 37 °C and 5% CO_2_ in a humidified incubator, seeded at 100,000 cells/mL and treated at 80% subconfluence. For the experiments adequate medium without phenol red was used.

### 4.3. Cell Viability Assay

To measure cell viability after treatment with DF extract for 24 and 72 h, the MTS assay was performed after seeding the cells in a 96 well plate. The commercially available CellTiter 96^®^ AQueous One Solution Cell Proliferation Assay from Promega Corporation (Mannheim, Germany) was used. The assay was conducted according to the supplier’s instructions. In brief, 20 µL MTS solution was put into each well and the plates were incubated for 1 h in the 37 °C incubator. Measurements were taken at 490 nm with the Elx800 (BioTek Instruments Inc., Bad Friedrichshall, Germany).

### 4.4. Cell Proliferation Assay

Cells were seeded at 100,000 cells/mL in a 24-well plate. The cell number was counted before seeding and after 24 h as well as 72 h of incubation. For the counting trypan blue solution was used (Merck). Cells were counted with an inverted light microscope Axiovert 100 (Carl Zeiss AG, Oberkochen, Germany).

### 4.5. VEGF ELISA

Cells were seeded into 24 well plates and treated with DF for three days, followed by the collection of the supernatant. A media exchange was conducted 24 h before supernatant collection. To determine the secreted VEGF amount the human VEGF DuoSet ELISA from R&D Systems (Wiesbaden, Germany) was used. The assay was performed according to the producer’s instructions. In parallel, the cell viability of the cells was determined to set it in relation to the secreted VEGF.

### 4.6. Real-Time PCR

RNA was isolated with the TRIZOL reagent (Invitrogen, Carlsbad, CA, USA), digested by DNase (Promega, Madison, WI, USA), and cDNA was synthesized using RevertAidTM H minus reverse transcriptase, (Thermo Scientific, Schwerte, Germany). Quantitative reverse transcription real time PCR (qRT-PCR) was performed using TaqMan primer probes (Applied Biosystems, Foster City, CA, USA) as described before [56]: glycerinaldehyde-3-phosphate-dehydrogenase (GAPDH) (Hs99999905_m1), VEGF-A (Hs_00173626_m1), FLT-1/VEGFR1 (Hs00176573_m1), KDR/VEGFR2 (Hs_00176676_m1). Fluorescent data were converted into cycle threshold (CT) measurements. ∆CT values of each sample were calculated as CT_gene of interes_t–CT_GAPDH_. A ∆CT value of 3.33 corresponds to one magnitude lower gene expression compared to GAPDH. Relative gene expression (∆∆CT values) was calculated with 2^(normalized CT non-stimulated – normalized CT stimulated)^ = n-fold of control.

### 4.7. Statistics

Four independent experiments per test were conducted at least. Diagrams, data tables and statistics were created with Microsoft Excel (Excel 2010, Microsoft, Redmond, WA, USA). The mean and standard deviation were calculated and pictured in the diagrams. Significances were calculated via One-Way ANOVA and multiple comparison tests with GraphPad PRISM 7 (GraphPad Software, Inc., San Diego, CA, USA, 2017). *p*-values under 0.05 were considered significant.

## 5. Conclusions

In contrast to other fucoidans, an extract from the brown alga *Dictyosiphon foeniculaceus* (DF extract) showed anti-proliferative effects on two tumor cell lines in a previous study. The aim of this work was, therefore, to evaluate potential antitumor effects of the DF extract on uveal melanoma (OMM-1) and glioblastoma cells (116-14, 118-14). For comparison, two healthy human cell lines (ARPE-19, SVGA) were included in the study. Tests for cell viability, VEGF secretion, proliferation and gene expression of VEGF-A, VEGFR1 and VEGFR2 were conducted after treatment with the DF extract. The extract decreased the cell viability and the proliferation of the OMM-1 cell line after 72 h, whereas neither the glioblastoma nor the healthy cells were affected. The gene expression of VEGFR1 was not influenced. The VEGFR2 mRNA expression was slightly decreased after 24 h in 116-14 cells, whereas the VEGF-A mRNA expression was increased in 118-14 cells after 72 h of stimulation. VEGF secretion by SVGA and 116-14 cells was increased after three days, but, conversely, that by ARPE-19 and OMM-1 was decreased, which indicates a potential anti-angiogenic effect. Thus, these initial experiments suggest that DF extract does not influence glioblastoma cells, but could exhibit anti-tumor effects on uveal melanoma.

Finally, the study with an DF extract demonstrated that not each brown alga contains considerable amounts of typical fucoidans, but these cell wall components may be “replaced” by other protein-associated glycans. Extracts from such algae do not exhibit activities as expected for typical fucoidans, but may have other activities as observed in this study.

## Figures and Tables

**Table 1:** Chemical characterization of extract from *Dictyosiphon feoniculaceus* ^1^.

Characteristics	Value
Molecular mass	193 ± 0.8 kDa
Sulfate content ^2^	8.8%
Fucose content	38.7%
Uronic acid	9.8 ± 0.6%
Total phenolic content	11.0 ± 3.2 μg GAE/mg ^3^
Protein content	24.1%

^1^ data from Bittkau et al., 2020 and Neupane et al., 2020 [24,25]; ^2^ apparent degree of sulfation amounts to only 0.1; ^3^ μg GAE/mg = gallic acid equivalents/mg.

## References

[1] Ohgaki H., Kleihues P. (2005). Epidemiology and etiology of gliomas. Acta Neuropathol..

[2] Gousias K., Markou M., Voulgaris S., Goussia A., Voulgari P., Bai M., Polyzoidis K., Kyritsis A., Alamanos Y. (2009). Descriptive Epidemiology of Cerebral Gliomas in Northwest Greece and Study of Potential Predisposing Factors, 2005–2007. Neuroepidemiology.

[3] Braun K., Ahluwalia M.S. (2017). Treatment of Glioblastoma in Older Adults. Curr. Oncol. Rep..

[4] Thakkar J.P., Dolecek T.A., Horbinski C., Ostrom Q.T., Lightner D.D., Barnholtz-Sloan J.S., Villano J.L. (2014). Epidemiologic and Molecular Prognostic Review of Glioblastoma. Cancer Epidemiol. Biomark. Prev..

[5] Kaliki S., Shields C.L. (2017). Uveal melanoma: Relatively rare but deadly cancer. Eye.

[6] Voelter V., Schalenbourg A., Pampallona S., Peters S., Halkic N., Denys A., Goitein G., Zografos L., Leyvraz S. (2008). Adjuvant intra-arterial hepatic fotemustine for high-risk uveal melanoma patients. Melanoma Res..

[7] Chattopadhyay C., Kim D.W., Gombos D.S., Oba J., Qin Y., Williams M.D., Esmaeli B., Grimm E.A., Wargo J.A., Woodman S.E. (2016). Uveal melanoma: From diagnosis to treatment and the science in between. Cancer.

[8] Willcox D.C., Scapagnini G., Willcox B.J. (2014). Healthy aging diets other than the Mediterranean: A focus on the Okinawan diet. Mech. Ageing Dev..

[9] Ford L., Stratakos A.C., Theodoridou K., Dick J.T.A., Sheldrake G.N., Linton M., Corcionivoschi N., Walsh P.J. (2020). Polyphenols from Brown Seaweeds as a Potential Antimicrobial Agent in Animal Feeds. ACS Omega.

[10] Dobrinčić A., Balbino S., Zorić Z., Pedisić S., Bursać Kovačević D., Elez Garofulić I., Dragović-Uzelac V. (2020). Advanced Technologies for the Extraction of Marine Brown Algal Polysaccharides. Mar. Drugs.

[11] Klettner A. (2016). Fucoidan as a Potential Therapeutic for Major Blinding Diseases—A Hypothesis. Mar. Drugs.

[12] Freitas R., Martins A., Silva J., Alves C., Pinteus S., Alves J., Teodoro F., Ribeiro H.M., Gonçalves L.M.D., Petrovski Ž. (2020). Highlighting the Biological Potential of the Brown Seaweed *Fucus spiralis* for Skin Applications. Antioxidants.

[13] Hsu H.-Y., Hwang P. (2019). Clinical applications of fucoidan in translational medicine for adjuvant cancer therapy. Clin. Transl. Med..

[14] Pozharitskaya O., Obluchinskaya E., Shikov A. (2020). Mechanisms of Bioactivities of Fucoidan from the Brown Seaweed Fucus vesiculosus L. of the Barents Sea. Mar. Drugs.

[15] Wang L., Oh J.Y., Kim Y.-S., Jeon Y.-J., Lee J.-S., Jeon Y.-J. (2020). Anti-Photoaging and Anti-Melanogenesis Effects of Fucoidan Isolated from *Hizikia fusiforme* and Its Underlying Mechanisms. Mar. Drugs.

[16] Fitton J.H., Dell’Acqua G., Gardiner V.-A., Karpiniec S., Stringer D.N., Davis E. (2015). Topical Benefits of Two Fucoidan-Rich Extracts from Marine Macroalgae. Cosmetics.

[17] Fitton J.H., Stringer D.N., Karpiniec S. (2015). Therapies from Fucoidan: An Update. Mar. Drugs.

[18] Dörschmann P., Bittkau K.S., Neupane S., Roider J., Alban S., Klettner A. (2019). Effects of Fucoidans from Five Different Brown Algae on Oxidative Stress and VEGF Interference in Ocular Cells. Mar. Drugs.

[19] Dörschmann P., Kopplin G., Roider J., Klettner A. (2019). Effects of Sulfated Fucans from Laminaria hyperborea Regarding VEGF Secretion, Cell Viability, and Oxidative Stress and Correlation with Molecular Weight. Mar. Drugs.

[20] Dörschmann P., Mikkelsen M.D., Nguyen T.T., Roider J., Meyer A.S., Klettner A. (2020). Effects of a Newly Developed Enzyme-Assisted Extraction Method on the Biological Activities of Fucoidans in Ocular Cells. Mar. Drugs.

[21] Rohwer K., Neupane S., Bittkau K.S., Galarza Pérez M., Dörschmann P., Roider J., Alban S., Klettner A. (2019). Effects of Crude Fucus distichus Subspecies evanescens Fucoidan Extract on Retinal Pigment Epithelium Cells-Implications for Use in Age-Related Macular Degeneration. Mar. Drugs.

[22] Bittkau K.S., Dörschmann P., Blümel M., Tasdemir D., Roider J., Klettner A., Alban S. (2019). Comparison of the Effects of Fucoidans on the Cell Viability of Tumor and Non-Tumor Cell Lines. Mar. Drugs.

[23] Greville R.K. (1830). Algae Britannicae, or Descriptions of the Marine and Other Inarticulated Plants of the British Islands, Belonging to the Order Algae; with Plates Illustrative of the Genera.

[24] Bittkau K.S., Neupane S., Alban S. (2020). Initial evaluation of six different brown algae species as source for crude bioactive fucoidans. Algal Res..

[25] Neupane S., Bittkau K.S., Alban S. (2020). Size distribution and chain conformation of six different fucoidans using size-exclusion chromatography with multiple detection. J. Chromatogr. A.

[26] Miyamoto N., De Kozak Y., Jeanny J.C., Glotin A., Mascarelli F., Massin P., Benezra D., Behar-Cohen F.F. (2006). Placental growth factor-1 and epithelial haemato–retinal barrier breakdown: Potential implication in the pathogenesis of diabetic retinopathy. Diabetologia.

[27] Ameratunga M., Pavlakis N., Wheeler H., Grant R., Simes J., Khasraw M. (2018). Anti-angiogenic therapy for high-grade glioma. Cochrane Database Syst. Rev..

[28] Gueven N., Spring K.J., Holmes S., Ahuja K.D.K., Eri R., Park A.Y., Fitton J.H. (2020). Micro RNA Expression after Ingestion of Fucoidan; A Clinical Study. Mar. Drugs.

[29] Azuma K., Ishihara T., Nakamoto H., Amaha T., Osaki T., Tsuka T., Imagawa T., Minami S., Takashima O., Ifuku S. (2012). Effects of Oral Administration of Fucoidan Extracted from Cladosiphon okamuranus on Tumor Growth and Survival Time in a Tumor-Bearing Mouse Model. Mar. Drugs.

[30] Venkatesan J., Singh S.K., Anil S., Kim S.-K., Shim M.S. (2018). Preparation, Characterization and Biological Applications of Biosynthesized Silver Nanoparticles with Chitosan-Fucoidan Coating. Molecules.

[31] Huang Y.-C., Lam U.-I. (2011). Chitosan/Fucoidan pH Sensitive Nanoparticles for Oral Delivery System. J. Chin. Chem. Soc..

[32] Jang B., Moorthy M.S., Manivasagan P., Xu L., Song K., Lee K.D., Kwak M., Oh J., Jin J.-O. (2018). Fucoidan-coated CuS nanoparticles for chemo-and photothermal therapy against cancer. Oncotarget.

[33] Kim H., Nguyen V.P., Manivasagan P., Jung M.J., Kim S.W., Oh J., Kang H.W. (2017). Doxorubicin-fucoidan-gold nanoparticles composite for dual-chemo-photothermal treatment on eye tumors. Oncotarget.

[34] Lee K.W., Jeong D., Na K. (2013). Doxorubicin loading fucoidan acetate nanoparticles for immune and chemotherapy in cancer treatment. Carbohydr. Polym..

[35] Lu K.-Y., Li R., Hsu C.-H., Lin C., Chou S.-C., Tsai M.-L., Mi F.-L. (2017). Development of a new type of multifunctional fucoidan-based nanoparticles for anticancer drug delivery. Carbohydr. Polym..

[36] Pawar V.K., Singh Y., Sharma K., Shrivastav A., Sharma A., Singh A., Meher J.G., Singh P., Raval K., Kumar A. (2019). Improved chemotherapy against breast cancer through immunotherapeutic activity of fucoidan decorated electrostatically assembled nanoparticles bearing doxorubicin. Int. J. Biol. Macromol..

[37] Wang P., Kankala R.K., Chen B., Long R., Cai D., Liu Y., Wang S. (2018). Poly-allylamine hydrochloride and fucoidan-based self-assembled polyelectrolyte complex nanoparticles for cancer therapeutics. J. Biomed. Mater. Res. Part A.

[38] Dithmer M., Kirsch A.-M., Richert E., Fuchs S., Wang F., Schmidt H., Coupland S.E., Roider J., Klettner A. (2017). Fucoidan Does Not Exert Anti-Tumorigenic Effects on Uveal Melanoma Cell Lines. Mar. Drugs.

[39] Lahrsen E., Liewert I., Alban S. (2018). Gradual degradation of fucoidan from Fucus vesiculosus and its effect on structure, antioxidant and antiproliferative activities. Carbohydr. Polym..

[40] Ehrig K., Alban S. (2014). Sulfated Galactofucan from the Brown Alga Saccharina latissima—Variability of Yield, Structural Composition and Bioactivity. Mar. Drugs.

[41] Deniaud-Bouët E., Kervarec N., Michel G., Tonon T., Kloareg B., Hervé C. (2014). Chemical and enzymatic fractionation of cell walls from Fucales: Insights into the structure of the extracellular matrix of brown algae. Ann. Bot..

[42] Schneider T., Ehrig K., Liewert I., Alban S. (2015). Interference with the CXCL12/CXCR4 axis as potential antitumor strategy: Superiority of a sulfated galactofucan from the brown algaSaccharina latissimaand Fucoidan over heparins. Glycobiology.

[43] Yuan Y.V., Walsh N.A. (2006). Antioxidant and antiproliferative activities of extracts from a variety of edible seaweeds. Food Chem. Toxicol..

[44] Cho M., Park G.-M., Kim S.-N., Amna T., Lee S., Shin W.-S. (2014). Glioblastoma-Specific Anticancer Activity of Pheophorbide a from the Edible Red Seaweed Grateloupia elliptica. J. Microbiol. Biotechnol..

[45] Karakaş C.Y., Şahin H.T., Inan B., Özçimen D., Erginer Y.Ö. (2019). In vitro cytotoxic activity of microalgal extracts loaded nano–micro particles produced via electrospraying and microemulsion methods. Biotechnol. Prog..

[46] Liao C.-H., Lai I.-C., Kuo H.-C., Chuang S.-E., Lee H.-L., Whang-Peng J., Lai G.-M., Lai G.-M. (2019). Epigenetic Modification and Differentiation Induction of Malignant Glioma Cells by Oligo-Fucoidan. Mar. Drugs.

[47] Lv Y., Song Q., Shao Q., Gao W., Mao H., Lou H., Qu X., Li X. (2012). Comparison of the effects of marchantin C and fucoidan on sFlt-1 and angiogenesis in glioma microenvironment. J. Pharm. Pharmacol..

[48] Zhao X., Guo F., Hu J., Zhang L., Xue C., Zhang Z., Li B. (2016). Antithrombotic activity of oral administered low molecular weight fucoidan from Laminaria Japonica. Thromb. Res..

[49] Pozharitskaya O.N., Shikov A.N., Faustova N.M., Obluchinskaya E., Kosman V.M., Vuorela H., Makarov V.G. (2018). Pharmacokinetic and Tissue Distribution of Fucoidan from Fucus vesiculosus after Oral Administration to Rats. Mar. Drugs.

[50] Pozharitskaya O., Shikov A., Obluchinskaya E., Vuorela H. (2019). The Pharmacokinetics of Fucoidan after Topical Application to Rats. Mar. Drugs.

[51] Tokita Y., Hirayama M., Nakajima K., Tamaki K., Iha M., Nagamine T. (2017). Detection of Fucoidan in Urine after Oral Intake of Traditional Japanese Seaweed, Okinawa mozuku (Cladosiphon okamuranus Tokida). J. Nutr. Sci. Vitaminol..

[52] Luyten G.P.M., Naus N.C., Mooy C.M., Hagemeijer A., Kan-Mitchell J., Van Drunen E., Vuzevski V., De Jong P.T.V.M., Luider T.M. (1996). Establishment and characterization of primary and metastatic uveal melanoma cell lines. Int. J. Cancer.

[53] Dunn K., Aotaki-Keen A., Putkey F., Hjelmeland L. (1996). ARPE-19, A Human Retinal Pigment Epithelial Cell Line with Differentiated Properties. Exp. Eye Res..

[54] Henriksen S., Tylden G.D., Dumoulin A., Sharma B.N., Hirsch H.H., Rinaldo C.H. (2014). The Human Fetal Glial Cell Line SVG p12 Contains Infectious BK Polyomavirus. J. Virol..

[55] Schweighardt J.T.S.B., Shieh J.T.C., Atwood W.J. (2001). CD4/CXCR4-independent infection of human astrocytes by a T-tropic strain of HIV-1. J. NeuroVirol..

[56] Adamski V., Hempelmann A., Flüh C., Lucius R., Synowitz M., Hattermann K., Held-Feindt J. (2017). Dormant glioblastoma cells acquire stem cell characteristics and are differentially affected by Temozolomide and AT101 treatment. Oncotarget.

